# Macroeconomic burden of tracheal, bronchial, and lung cancers in Asia

**DOI:** 10.3389/fpubh.2026.1943102

**Published:** 2026-10-12

**Authors:** Long Chen, Haoyang Hu, Jiuhe Wang, Jinglian Qu

**Affiliations:** 1 Guizhou University of Traditional Chinese Medicine, Guiyang, China; 2 Medical School of Chinese PLA, Beijing, China; 3 Inner Mongolia Autonomous Region Hospital of Traditional Chinese Medicine, Branch of National Clinical Research Center for Chinese Medicine Cardiology, Hohhot, China

**Keywords:** Asia, DALY, macroeconomic burden, tracheal, bronchial, and lung cancers, VLW

## Abstract

**Background:**

Although Asia shoulders a considerable burden of tracheal, bronchial, and lung (TBL) cancers, the associated macroeconomic impact has yet to be adequately investigated.

**Methods:**

Using 2023 data from the Global Burden of Disease Study and the World Bank, we applied the value of a statistical life year (VSLY) method to estimate the economic welfare losses attributable to TBL cancers in Asian countries. We further analyzed the temporal trends, sex and age-specific disparities, and the drivers of economic inequality and projected the burden trends to 2050.

**Results:**

In Asia, the value of lost welfare (VLW) due to these cancers reached USD 3.28 (95% UI, 2.6301–4.0572) trillion, equivalent to 4.18% (95% UI, 3.35–5.17) of the regional GDP. Temporal trend analyses revealed a consistent upward trajectory in both absolute VLW and its GDP share. Sex-stratified analyses showed that the burden was significantly higher in male populations, and age-specific peaks occurred at 70–74 years for both sexes. Health inequality metrics indicated a pronounced widening of relative disparities over time. Projection models forecast a continued increase in absolute VLW, whereas its proportional contribution to GDP is expected to plateau.

**Conclusion:**

These findings establish that TBL cancers impose a substantial and inequitably distributed macroeconomic burden across Asia. The pronounced sex and region-specific disparities underscore the urgent need for evidence-based, geographically targeted intervention strategies. Prioritizing the targeted deployment of resources in high-burden zones is critical to mitigating economic losses and improving survival outcomes in the region.

## 1 Introduction

TBL cancers pose a major global public health burden. As the leading cause of cancer-related mortality, TBL cancers accounted for 2.04 million deaths worldwide in 2023, with a DALY rate of 505 (95% UI, 456–550) per 100,000 population (1). The burden is particularly pronounced in Asia, which accounts for over 60% of global TBL cancers cases and deaths, reflecting a complex interplay of demographic transitions, environmental exposures, and socioeconomic disparities across the region (2). Health system capacity and accessibility to medical services are critical determinants of lung cancer prognosis; however, both vary considerably among Asian countries (3). High-income nations have made substantial progress in TBL cancers management through comprehensive cancer control programs, including organized screening initiatives. In contrast, many low- and middle-income countries continue to face formidable challenges in diagnostic infrastructure, treatment availability, and palliative care services (4). These disparities in resource availability directly contribute to marked differences in stage distribution, therapeutic strategies, and ultimately survival outcomes across countries and regions, thereby exacerbating health inequalities along socioeconomic gradients. Despite considerable advances in diagnostic techniques and treatment modalities over recent decades, overall prognosis remains poor, with global 5-year survival rates below 15% (5).

Globally, TBL cancer ranks among the leading causes of premature mortality and disability, with socioeconomic consequences that extend well beyond the direct health losses to individuals. At the patient level, the disease not only compromises quality of life and functional capacity but also reduces labor supply and income, thereby directly undermining financial self-sufficiency (6). Nevertheless, the economic burden of TBL cancer is not confined to the affected individuals. At the household level, the high costs of diagnosis, treatment, and care frequently result in catastrophic health expenditures, particularly in regions with limited health insurance coverage, where patient families are highly susceptible to impoverishment or re-impoverishment due to illness (7). Concurrently, the diversion of household funds from children’s education to cover medical expenses may further perpetuate intergenerational poverty (8). At the health-system level, the substantial resources required for TBL prevention and treatment inevitably crowd out investments in other public health priorities, such as infectious disease control, maternal and child health, and chronic disease management, incurring considerable opportunity costs (9). At the national level, the loss of human capital attributable to premature death and disability among working-age populations imposes a persistent drag on macroeconomic growth (10). In summary, the socioeconomic impact of TBL cancer is not a unidimensional disease burden but rather a cascade of interlinked effects involving household financial vulnerability, health-system resource allocation patterns, and macroeconomic development potential.

Although the disease burden of TBL cancer has received considerable attention, its macroeconomic consequences—particularly in Asia, which accounts for the highest proportion of global TBL cancer cases and deaths—remain insufficiently systematically assessed. Liu et al. (11) conducted the first global assessment of the macroeconomic burden of TBL cancer using the VLW framework based on GBD 2021 data. While that study provided a critical baseline for understanding the global economic impact, its analysis was restricted to a cross-sectional estimate for a single year (2021) at the global level, without addressing long-term temporal trends, age-specific distributions, interregional inequalities, or future burden projections. Given the accelerating population aging, the epidemiological transition from communicable to non-communicable diseases in low-income countries, and the substantial disparities in socioeconomic development across countries, the macroeconomic impact of TBL cancer in this region may exhibit unique spatiotemporal evolution patterns. Therefore, building upon the seminal work of Liu et al., the present study focuses on Asia, using the same VLW framework with GBD 2023 data to systematically estimate the welfare losses attributable to TBL cancer across 46 Asian countries from 1990 to 2023 and to project the burden through 2050, with the aim of providing robust evidence to inform equitable resource allocation and global health policy formulation (12).

## 2 Methods

### 2.1 Data sources

We accessed GBD 2023 through the GHDx Results Tool, a database covering 375 diseases in 204 countries/territories that provides the latest burden estimates and serves as an essential resource for public health research (13). We extracted DALYs for Asian countries from 1990 to 2023. All estimates, except percentage changes, are presented with their 95% uncertainty interval (UI). The point estimates are means of 500 draws, and the UI correspond to the 2.5th and 97.5th percentiles of the draw distribution (14). In the ICD-10 coding system, lung cancer (C33-C34) includes tracheal and bronchial cancers, reflecting this comprehensive classification (15). Gross domestic product (GDP) and GDP per capita were obtained from the World Development Indicators database, and the baseline value of a statistical life was derived from the 2023 estimate of $13.2 million by the U.S. Department of Transportation. These economic variables were adjusted for purchasing power parity and reported in 2023 international dollars to remove price-level discrepancies, thereby improving the credibility of cross-country comparisons. The VLW/GDP ratio, which quantifies the macroeconomic burden of TBL cancers relative to national productivity, standardizes cross-economy comparisons, and a higher value signals a disproportionate impact versus productive capacity.

### 2.2 Estimation of VLW

The VLW is a standardized model used to quantify macroeconomic losses by integrating DALYs and the value of a statistical life. VSL is defined as the amount individuals are willing to pay to reduce mortality risk, reflecting the marginal rate of substitution between personal wealth and mortality risk (i.e., the trade-off between wealth and the risk of death). The VLW model further quantifies both market losses (e.g., income loss) and non-market losses (e.g., health status value and leisure time) attributable to specific diseases by incorporating disease-specific information (16). We applied the VSL framework to monetize DALYs, using the VSLY as the willingness-to-pay metric. Country-specific VSL values were transferred using the benefit transfer method, as detailed in earlier study, with the formula (17): *VSL_{c,t}_ = VSL_{USA,2023}_ × (GDP_pc{c,t}_/ GDP_pc{USA,2023}_)^^{ie}^,* where *VSL_{USA,2023}_* is the US VSL at 2023 prices; *GDPpc_{USA,2023}_* is the US GDP per capita; *GDPpc_{c,t}_* is the per-capita GDP for country c in year t; and ie is the income elasticity (set to 1.0 in the primary analysis). For each country, VSLY was derived by dividing VSL by half of the national life expectancy, approximating average remaining lifespan, under the assumption that DALYs are uniformly distributed over the remaining lifespan. The total welfare loss for each country was estimated as *VLW_{c,t}_ = DALYs_{c,t}_ × VSLY_{c,t}_*, where DALYs_{c,t}_ are the disability-adjusted life years and *VSLY_{c,t}_* is the country-specific VSLY. All monetary figures were converted to 2023 international dollars using PPP factors, with GDP and per-capita GDP from the World Bank Open Data (constant 2023 international dollars). To assess the relative economic burden of TBL cancer, we computed the *VLW/GDP* ratio: *VLW/GDP ratio_{c,t}_ = (VLW_{c,t}_/GDP_{c,t}_) × 100%*. This relative measure eliminates the effect of absolute economic size and enables comparisons across different income regions.

This study employed the slope index of inequality (SII) and the concentration index, as defined by the World Health Organization (WHO), to measure absolute and relative inequalities in disease burden. The SII was derived by regressing DALY rates against the Socio-demographic Index (SDI), using the midpoint of the cumulative population distribution ranked by SDI. To better control for bias and heterogeneity, we applied robust regression models (rlm) instead of ordinary linear regression (lm) in the health inequality analysis; robust regression reduces sensitivity to outliers and minimizes bias due to data heterogeneity or extreme values, thus providing more accurate estimates of health inequality. The concentration index was computed by matching the cumulative proportion of DALYs against the cumulative population distribution ranked by SDI and numerically integrating the area under the Lorenz curve (18).

Future burden was projected from 2024 to 2050 using a Bayesian age-period-cohort model implemented in the BAPC package with integrated nested Laplace approximation through R-INLA. For each outcome and sex–location combination, observed age-specific event counts for 1990–2023 and corresponding age-specific population denominators for 1990–2050 were arranged into age-period matrices. Counts were modeled using a Poisson likelihood with population as the denominator. The matrices contained 20 five-year age groups, ranging from younger than 5 years to 90–94 years, plus a final group of 95 years and older, and were supplied to APCList with a 5-year age-group interval.

The log rate was represented by age, period, and cohort effects together with an overdispersion component. Age and cohort effects were specified as second-order random walks, the period effect as a first-order random walk, and overdispersion as an independent and identically distributed effect. Precision parameters for the age, period, and cohort components were assigned log-Gamma priors with parameters c(1, 0.00005); the overdispersion precision used a log-Gamma prior with parameters c(1, 0.005). Projections were generated with npredict = 27 for 2024–2050 and retro = TRUE. Age-standardized rates were derived using standard-population weights restricted to the modeled age groups and renormalized to sum to one. We extracted posterior age-specific rates and numbers, age-standardized rates, and projected total numbers; rates were reported per 100,000 population, and 95% uncertainty intervals were calculated as the posterior mean plus or minus 1.96 posterior standard deviations. All analyses were performed in R version 4.5.0 using BAPC version 0.0.36 and R-INLA version 24.12.11. Data processing and visualization used dplyr version 1.1.4, tidyr version 1.3.1, purrr version 1.0.4, and ggplot2 version 3.5.2. This study complies with the Consolidated Health Economic Evaluation Reporting Standards to promote methodological transparency and reproducibility.

## 3 Results

In 2023, the total VLW attributed to TBL cancers in Asia reached USD 3.28(95% UI, 2.6301–4.0572) trillion, equivalent to 4.18% (95% UI, 3.35–5.17) of the region’s aggregate GDP. Marked heterogeneity was observed across subregions: East Asia bore the largest share, contributing USD 2.0114 (95% UI, 1.6488–2.461) trillion (61.3% of the Asian total), followed by high-income Asia-Pacific with 14.6%. Southeast Asia and West Asia accounted for comparable proportions of 8.99 and 7.93%, respectively, whereas South Asia and Central Asia contributed smaller shares of 6.04 and 1.11% (Figure 1A; Supplementary Table S1). Substantial disparities were also evident at the national level. China recorded the highest VLW (USD2, 011.4 billion, 95% UI: 1,648.8–2,461.0), followed by Japan (USD354.1 billion, 95% UI: 297.7–400.5) and Türkiye (USD 174.0 billion, 95% UI: 116.2–224.7). In contrast, the lowest burdens were observed in Timor-Leste (USD 90 million, 95% UI: 60–140 million), Bhutan (USD 120 million, 95% UI: 80–180 million), and the Maldives (USD 130 million, 95% UI: 100–170 million), yielding a more than 20,000-fold difference between the highest and lowest country estimates. Notably, China alone contributed over 61.3% of the total Asian VLW. High-income nations particularly Japan, the Republic of Korea, and Singapore ranked among the top contributors. Moreover, countries with heavy burdens spanned a wide spectrum of SDI levels, ranging from high SDI (Japan and Korea) to low-middle SDI (India and Vietnam), suggesting that the economic impact of TBL cancers is not determined solely by economic development but is likely shaped by a confluence of multiple factors (Figure 2A; Supplementary Table S2).

**Figure 1:**
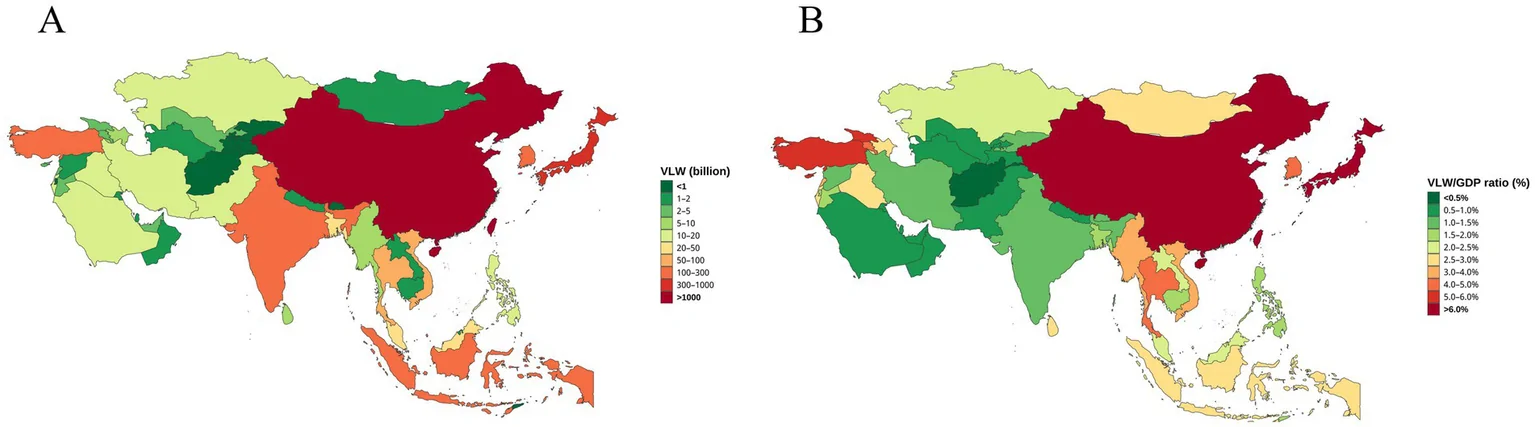
**(A)** VLW from TBL cancer in Asia, 2023. **(B)** VLW from TBL cancer as a share of GDP in Asia, 2023.

**Figure 2:**
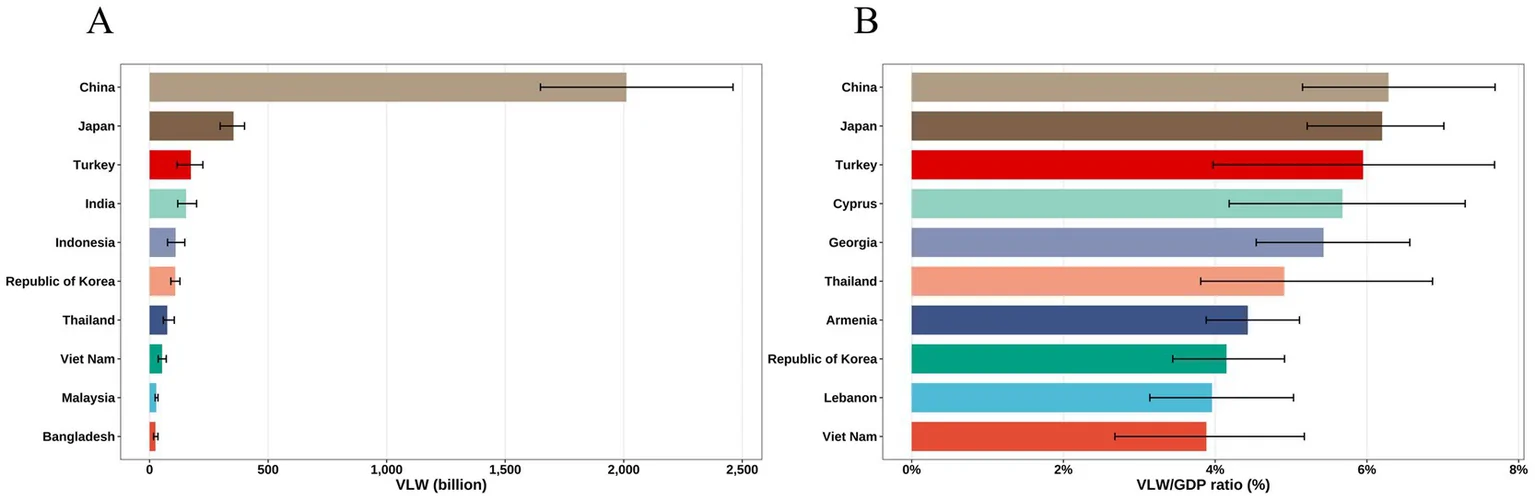
**(A)** Top 10 countries in Asia by VLW. **(B)** Top 10 Asian countries by VLW as a share of GDP.

The ratio of VLW to GDP also exhibited marked geographical heterogeneity across Asian countries. China (6.28, 95% UI, 5.15–7.69%), Japan (6.20, 95% UI, 5.21–7.01%), and Türkiye (5.95, 95% UI, 3.97–7.68%) ranked among the highest, followed by Cyprus (5.68, 95% UI, 4.18–7.30%), Georgia (5.43, 95% UI, 4.54–6.56%), and Armenia (4.43, 95% UI, 3.88–5.11%), which also registered relatively high values. At the opposite end, Afghanistan (0.30, 95% UI, 0.19–0.49%), Qatar (0.50, 95% UI, 0.33–0.79%), and Oman (0.60, 95% UI, 0.42–0.85%) showed the lowest ratios. It is worth noting that Türkiye’s estimate had a notably wide uncertainty interval, suggesting that the point estimate may be less robust and should be interpreted with caution (Figure 1B; Supplementary Table S3). When countries were re-ordered by the VLW/GDP metric, the burden profile of TBL cancers in Asia underwent substantial changes. First, although East Asian countries continued to occupy leading positions in terms of relative burden, the gaps among them narrowed considerably: China, Japan, and Türkiye took the top three places, with ratios clustering closely. Notably, Japan’s VLW in absolute terms was only one-sixth of China’s VLW; however, its VLW/GDP ratio was nearly identical, implying that the economic erosion caused by lung cancer is no less consequential for Japan’s national economy. Second, several smaller economies moved markedly upward in the relative ranking, exposing the vulnerability of their macroeconomic systems. Although countries such as Cyprus, Georgia, Armenia, and Lebanon have relatively low absolute burdens, their VLW/GDP ratios exceed those of developed industrial nations such as the Republic of Korea, largely owing to their limited GDP sizes. This phenomenon was particularly prominent in West Asia. In contrast, Saudi Arabia, the United Arab Emirates, Kuwait, Oman, and Qatar, despite having relatively high SDI levels, all fell into the lowest tier, with VLW/GDP ratios ranging from 0.50 to 0.80%. Furthermore, India and Pakistan may have had their relative burdens substantially diluted by their large economic aggregates: Although their absolute VLWs ranked 4th and 18th in Asia, respectively, their VLW/GDP rankings dropped to 32nd and 41st (Figure 2B; Supplementary Table S3).

From 1990 to 2023, the absolute VLW attributable to TBL cancers in Asia increased from approximately US$218.8 (95% UI: 183.37–269.74) billion to US$3.28 (95% UI: 2.6301–4.0572) trillion, representing a roughly 15-fold rise. A notable uptick occurred in 2020. Sex-stratified analysis revealed that male populations consistently bore the major share of the burden, whereas female populations showed a slightly faster growth rate (Figure 3A; Supplementary Table S4). Over the same period, the VLW/GDP ratio climbed from 1.48% (95% UI: 1.24–1.82%) to 4.18% (95% UI: 3.35–517%), reflecting a steadily mounting relative burden on the Asian economy, with an accelerated upward trajectory after 2020 (Figure 3B; Supplementary Table S4).

**Figure 3:**
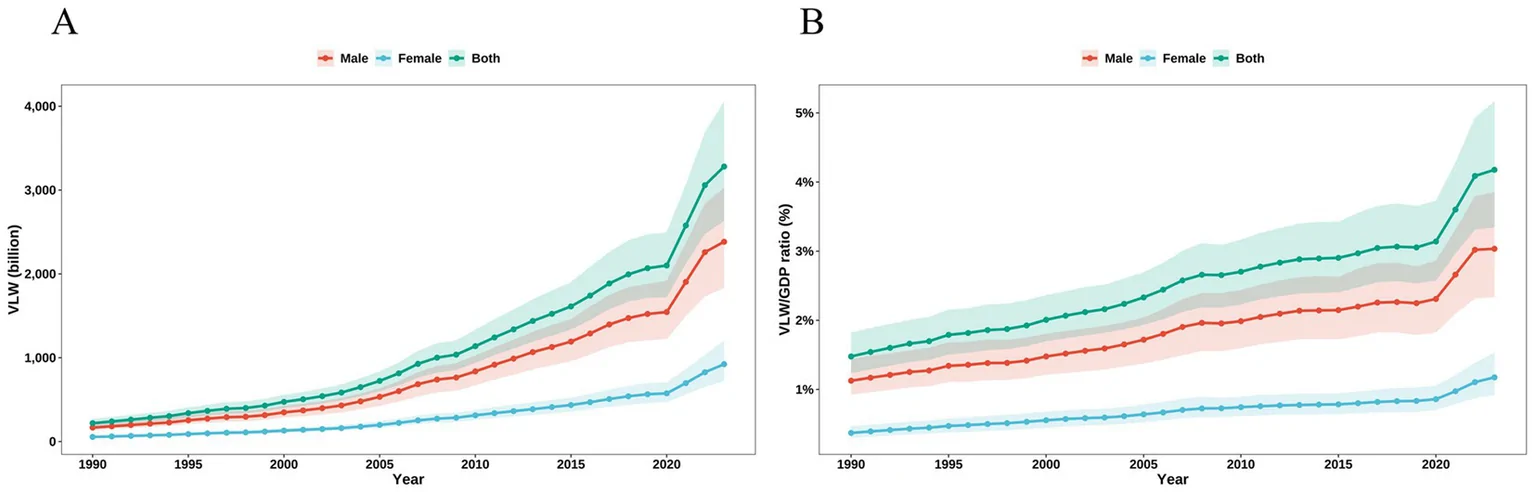
**(A)** Trends in the VLW, 1990–2023. **(B)** Trends in the VLW as a share of GDP, 1990–2023.

In 2023, the VLW attributable to TBL cancers in Asia was nil before age 15, emerged in the 15- to 19-year age group, and rose steeply with advancing age. For male populations, the VLW peaked at 70–74 years and declined thereafter; for female populations, the peak also occurred at 70–74 years, but the subsequent decline was more gradual. Male populations had significantly higher VLW than female populations across all age groups, with the notable exception of the oldest-old (≥95 years), where female VLW surpassed that of male populations (Figure 4A; Supplementary Table S5). The VLW/GDP ratio exhibited a similar age-related pattern (Figure 4B; Supplementary Table S6). Overall, the VLW due to lung cancer was concentrated predominantly in middle-aged and older populations, with male populations bearing the vast majority of the economic burden.

**Figure 4:**
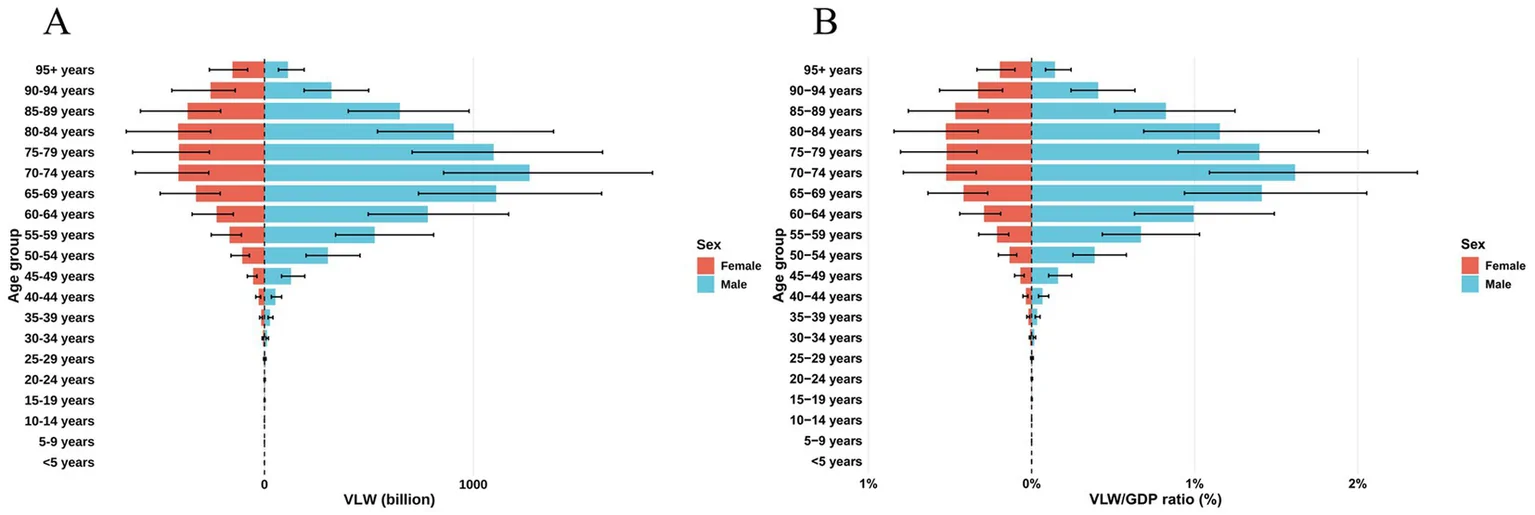
**(A)** Sex and age analysis of the VLW, 2023. **(B)** VLW as a share of GDP by sex and age, 2023.

Health inequality analysis revealed an overall worsening of the VLW inequality pattern for TBL cancers in Asia in 2023. The SII rose from 2.66 in 1990 to 6.83 in 2023, corresponding to a 157.3% increase, indicating a marked widening of the relative gap between the highest and lowest socioeconomic groups. Although the uncertainty intervals were relatively wide, the upward trend was clear. Over the entire observation period, the SII fluctuated but generally followed an ascending trajectory, suggesting that while the absolute disparity in TBL cancer burden across regions with different SDI levels widened on the whole, certain periodic features were also evident (Figures 5A, B; Supplementary Table S7).

**Figure 5:**
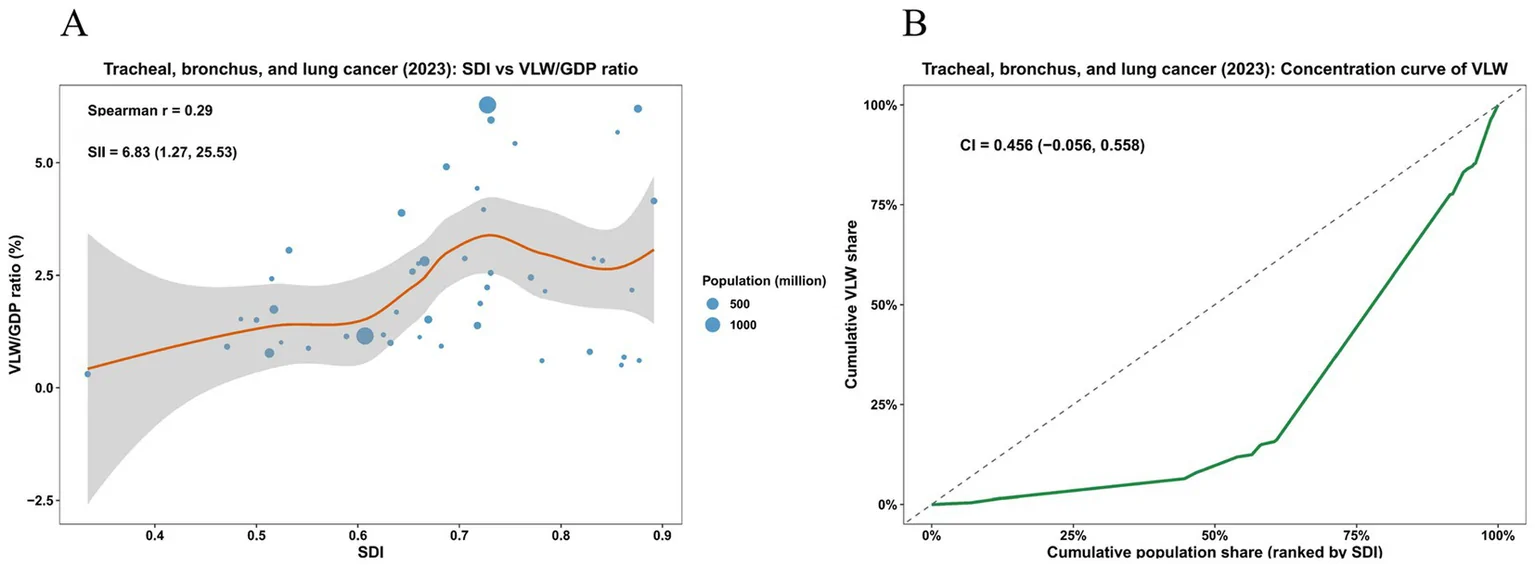
Health inequality analysis of TBL cancer in Asia, 1990–2023. **(A)** SII index. **(B)** CI index.

Projections indicate that the absolute VLW will continue to increase steadily from 2024 to 2050, reaching approximately $12.13 trillion (95% UI: 4.17–20.09) by 2050, representing an approximately 270% increase from the 2023 level. However, the VLW-to-GDP ratio exhibits a transient decline in 2025, followed by a gradual rise to 5.76% (95% UI: 4.79–6.73%) by 2050. This projected trajectory—characterized by sustained absolute growth and an initial dip followed by a subsequent rebound in the relative share—suggests that while short-term economic recovery may partially dilute the relative intensity of the disease burden, in the long term, the macroeconomic strain imposed by lung cancer on Asia will continue to intensify, driven by population ageing and the escalating disease burden (Figure 6; Supplementary Table S8).

**Figure 6:**
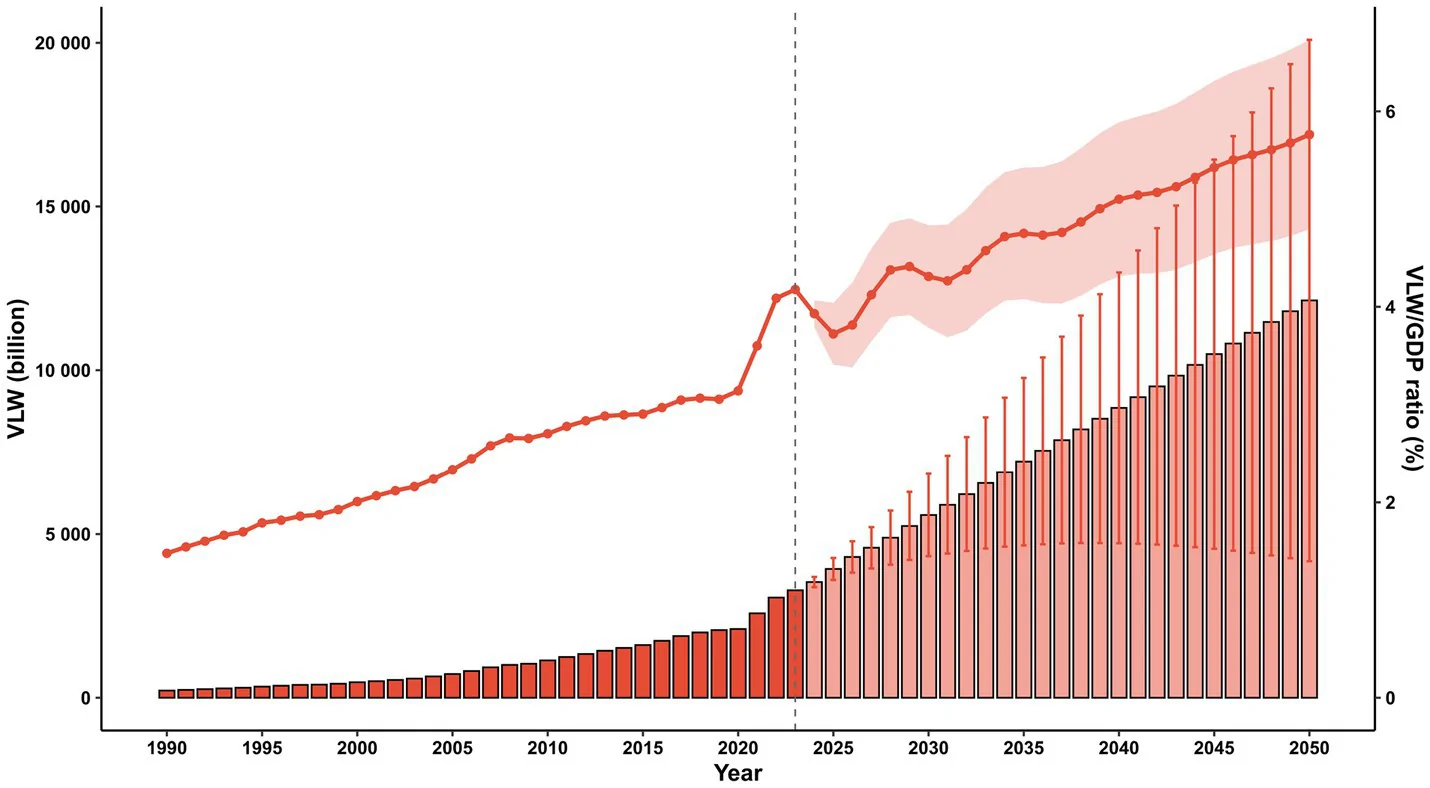
Forecast of VLW and VLW/GDP due to TBL cancer in Asia, 2024–2050.

## 4 Discussion

TBL cancer is the second most common malignancy globally and the leading cause of cancer-related mortality (19). Its disease burden has been driven by factors including tobacco smoking, population ageing, and environmental pollution, with a sustained upward trend (20) and substantial regional disparities (21). Systematic quantitative investigations into the overall economic burden of TBL cancer in Asia and its long-term temporal evolution remain scarce. This gap limits a comprehensive understanding of the socioeconomic impact of TBL cancer and undermines the scientific basis for resource allocation decisions. To address this, the present study systematically analyzed the disease-related economic burden of TBL cancer in Asia from 1990 to 2023, with a focus on age-specific distribution, temporal trends, driving factors, and future projections, aiming to provide evidence-based support for the development of region-specific and precision prevention strategies tailored to Asia’s diverse socioeconomic contexts. Our findings reveal a sustained and rapid increase in the economic burden of TBL cancer in Asia over the past three decades, with the absolute value rising approximately 15-fold and the share of GDP increasing roughly 1.82-fold, indicating a progressively deepening macroeconomic erosion by this disease. Further analysis showed marked disparities in economic burden indicators across Asian countries, with a pronounced concentration in East Asia, suggesting that absolute economic losses and relative economic impacts do not simply move in tandem.

### 4.1 Core drivers and underlying determinants of the economic burden in East Asia

The sustained increase in the economic burden of TBL cancers in East Asia results from the interplay of three interacting forces: expanding patient populations, technological upgrading of medical care with associated cost-structure shifts, and pharmaceutical pricing along with policy factors. First, the rapid expansion of the patient base is the fundamental driver. Smoking and ambient air pollution, as the foremost risk factors, directly elevate the incidence and mortality of lung cancer, contributing the largest share of DALYs (22). Concurrently, population aging amplifies the disease burden, while the marked rise in female lung cancer incidence and its trend toward younger age at onset further enlarge the affected population (23). Second, advances in medical technology, while improving therapeutic outcomes, have reshaped cost structures and driven up both per-episode and overall treatment expenses. The introduction of innovative agents—such as targeted therapies and immunotherapies—and the widespread adoption of minimally invasive procedures such as video-assisted thoracoscopic surgery, although enhancing quality of life, entail high prices and costly consumables, directly contributing to increasing hospitalization expenditures (24). Inpatient costs are transitioning from a “drug-dominant” to a “technology and consumables dominant” pattern, with surgical and material fees becoming primary growth drivers. Finally, drug pricing, health insurance policies, and prevention investments profoundly affect the economic burden. Although government regulation of drug prices may help contain costs, it can also induce substitution effects. Inequities in treatment efficiency and insurance benefits among lung cancer patients remain pronounced, necessitating expanded medical insurance coverage to alleviate financial strain (25). Of note, effective prevention measures can yield substantial economic returns; for instance, achieving ambient air quality standards could prevent a considerable number of lung cancer cases and save direct medical expenditures (26). The above experiences offer valuable references for other Asian countries confronting similar epidemiological transitions and resource constraints: On the one hand, stratified and phased prevention strategies should be established according to each country’s level of economic development and disease spectrum; on the other hand, lung cancer control should be integrated into comprehensive chronic disease management systems, with due attention to cost-effectiveness assessments across the entire screening-diagnosis-rehabilitation continuum, so as to maximize health gains under limited resources.

### 4.2 Multidimensional determinants of sex disparities

In the economic burden of TBL cancers in Asia, male populations consistently exhibit higher VLW and VLW/GDP ratios than female populations across all age groups, with the gap slightly widening with advancing age. The observed disparities are attributable to multiple factors. Smoking is universally recognized as the leading risk factor for TBL cancers; despite notable progress in global tobacco control and declining smoking rates in many countries, smoking prevalence and secondhand smoke exposure remain stubbornly high among male populations and in rural areas, particularly in China (27). Occupational segregation is another important contributor—gender-based occupational segregation is especially pronounced in Asia, with men disproportionately employed in high-risk industries such as mining and construction, involving prolonged exposure to carcinogens including asbestos and radon. In addition, delayed health-seeking behavior among male populations may lead to later-stage diagnoses and poorer prognoses. Although active smoking rates are lower among female populations, indoor air pollution from cooking and heating plays a substantial role in lung cancer development. Furthermore, hormonal and genetic interactions are implicated; environmental carcinogens may exert effects through estrogen receptor signaling pathways (e.g., ERα, ERβ, and GPER), thereby promoting lung adenocarcinoma a subtype that is disproportionately more common in female than in male populations (28). At the same time, sex-specific barriers severely impede the entire diagnostic and therapeutic pathway for female TBL cancers patients: the average delay from symptom onset to diagnosis extends to 7.4 months, with financial constraints, geographic inaccessibility, and health system limitations constituting major obstacles (29); social stigmatization and implicit biases within the healthcare system further compound gaps in the continuum of TBL cancers care for women, adversely affecting clinical outcomes (30). However, with rising smoking rates and increasing occupational exposures among women in some countries, female TBL cancer incidence is rapidly escalating, highlighting the urgent need for sex-sensitive prevention and control strategies (31).

### 4.3 Age-specific distribution patterns and regional drivers

The prevalence of TBL cancer is notably high among individuals aged 70 years and older. Beyond tobacco smoking, the core drivers include demographic shifts driven by accelerated population aging—both the changing age structure and the expanding absolute size of the older adult population, as age itself is the strongest risk factor for TBL cancer, with cumulative DNA damage and immunosenescence intensifying over the life course. Regional disparities between resource‑limited settings with delayed diagnosis and undertreatment in low‑ and middle-SDI regions and more effective prevention and control in high‑SDI areas further shape the current pattern of disease burden among the older adult (32). As global aging continues to deepen, the overall burden of TBL cancer in older adults is expected to rise steadily, even if current levels of non-smoking risk factors remain unchanged.

### 4.4 Non-linear relationship between socioeconomic development and economic burden

The relationship between socioeconomic development and the disease burden of TBL cancers is not a simple negative linear one; rather, the distribution pattern across Asian countries exhibits a complex socioeconomic gradient. Previous studies have generally anticipated that disease burden should decline as socioeconomic conditions and healthcare infrastructure improve (33). However, our data-driven analysis reveals a distinctly different pattern: Countries with high burden span multiple SDI levels, indicating that the economic burden of lung cancer is not determined solely by economic development but is also shaped by population size, smoking prevalence, air pollution, and other contextual factors. This non-linear association implies a multilayered, multi-pathway interaction mechanism between socioeconomic progress and TBL cancer-related economic burden.

The impact of industrialization on TBL cancers displays a pronounced dual character. On the one hand, environmental pollution arising from industrial activities, particularly exposure to ambient fine particulate matter (PM₂.₅), has been firmly established as a significant risk factor for lung cancer; a meta-analysis showed that each 10 μg/m^3^ increase in PM₂.₅ concentration is associated with a 10 and 17% increase in lung cancer incidence and mortality, respectively, among never-smokers, suggesting that the cumulative environmental load during rapid industrialization in East Asia may be a major driver of the persistently high disease burden in this region (34). On the other hand, post-industrial environmental governance and health promotion policies have begun to show positive effects—for example, after implementing air quality management measures, South Korea observed declines in certain short-term mortality outcomes related to air pollution, indicating that improved air quality holds potential for long-term benefits in TBL cancer control and underscoring the critical role of the timing and intensity of environmental policy interventions in the evolution of disease burden (35). The relationship between medical technological advancement and disease burden is equally complex. High-income countries possess state-of-the-art diagnostic and therapeutic modalities; however, widespread early screening may inflate prevalence estimates; for instance, Japan’s relatively high prevalence is partly attributable to its well-established cancer registration system and universal health checkup programs (36). In contrast, the true disease burden in low-income countries is often substantially underestimated due to underdiagnosis—for example, in India, cancer registries cover less than 15% of the urban population and only approximately 1% of the rural population (37), with national coverage estimated at roughly 10–11% (38). The considerable disparities in surveillance intensity and diagnostic capacity across countries constitute a major source of bias in cross-national comparisons of TBL cancer burden. In countries that have adopted low-dose CT screening (e.g., Japan and South Korea), reported increases in incidence and prevalence may more accurately reflect enhanced case detection rather than a genuine rise in underlying risk, and previous studies have indicated that a substantial proportion of lung cancers detected through intensive CT screening may represent overdiagnosis, thereby inflating prevalence estimates (39). Conversely, the lower estimates observed in parts of South Asia and Central Asia are more likely attributable to incomplete registration, underdiagnosis, and weak surveillance systems that leave a large number of cases undetected (40). Nevertheless, the residual differences across countries still remind us that sustained investment in high-quality, population-based cancer surveillance systems—especially in low- and middle-income regions—remains of paramount practical importance.

### 4.5 Structural factors driving worsening health equity

The deterioration of health equity is rooted in structural problems. In the context of globalization, the unequal distribution of medical resources has intensified: Innovative therapies are predominantly concentrated in high-income countries, making them largely inaccessible to low-income patients (41); even where health insurance coverage exists, urban–rural disparities remain substantial (e.g., 5-year cancer survival rates in China are 46.7% in urban areas versus 33.6% in rural areas) (42); and disparities in health literacy further weaken prevention awareness among disadvantaged groups (43). Subnational analyses indicate that, even within the same country (e.g., India), although the absolute gap in state-level burden is smaller than that between countries, its rate of increase is 2.8 times that of cross-country disparities, signaling that within-country inequalities are expanding rapidly. At the environmental level, low-SDI regions often become destinations for pollution-intensive industries (44) and target markets for tobacco companies (45), creating a “pollution haven” effect. Novel features of health inequity under globalization include that the dividends of innovation benefit only a few, while low-income regions grapple with a dual burden of communicable and non-communicable diseases (46).

### 4.6 Future trends and optimization of prevention strategies

Projections based on the BAPC model indicate that by 2050, all major disease burden indicators for TBL cancers in Asia will continue to rise. In the absence of timely and effective interventions, lung cancer is poised to become an increasingly serious public health challenge in the region. Addressing this trajectory requires the establishment of a multi-tiered, multidimensional comprehensive prevention and control system. At the primary prevention level, tobacco control remains the most cost-effective fundamental strategy. The six core measures proposed by the WHO—monitoring tobacco use, protecting the public from secondhand smoke, providing cessation support, warning about tobacco hazards, enforcing smoking bans, and raising tobacco taxes—have achieved notable success in multiple countries (47). Concurrently, environmental management warrants equal attention; stricter air quality standards should be enacted and the transition to clean energy accelerated to reduce population exposure to carcinogens such as fine particulate matter. At the secondary prevention level, standardized implementation of low-dose computed tomography screening should be promoted, alongside the development of artificial intelligence-based risk stratification systems to optimize the identification of high-risk individuals and improve early detection rates of lung cancer (48). At the tertiary prevention level, dissemination of standardized diagnostic and treatment protocols is needed, together with enhanced accessibility to palliative care and rehabilitation services, so as to improve the quality of life and overall prognosis for patients with advanced disease. In summary, the projected upward trajectory of the lung cancer burden in Asia over the next decade serves as a stark warning: only through a government-led, multisectoral, population-wide comprehensive strategy can the escalating impact of this disease on socioeconomic development and population health be effectively contained.

## 5 Limitations and future directions

Several limitations of this study warrant consideration. First, our estimates rely on data from the GBD 2023 study, which are derived from modeling and secondary input sources. The quality of primary data varies considerably across low- and middle-income countries, and the inherent uncertainty in DALY estimates may affect the precision of our VLW results. Second, although the VSLY approach facilitates cross-national comparability, its key parameters including the VSLY and associated economic valuations are predominantly derived from high-income settings and may not fully capture cross-regional heterogeneity in cultural contexts and institutional arrangements, potentially constraining the applicability and interpretability of our findings in specific local contexts. Third, our analysis focused solely on DALY-related welfare losses and did not account for non-fatal or indirect effects of TBL cancers, nor did it capture specific information on indirect economic or intangible costs. Consequently, the VLW estimates presented in this study represent conservative figures. Future work ought to enhance country-level micro-cost data collection and combine health economic models with behavioral policy research.

## 6 Conclusion

In 2023, the economic burden attributable to TBL cancer in Asia reached a substantial level, with its share of regional GDP rising markedly compared with previous years. However, marked regional disparities persist. When assessed in terms of relative economic impact, some smaller economies exhibited a burden ratio even higher than that of developed industrial nations such as the Republic of Korea, whereas in several populous countries, the relative burden was substantially diluted by their large economic output. In terms of sex and age distribution, the burden peaked in older age groups among male populations, with male populations bearing a higher burden than female populations across all age groups, except for the oldest-old category, where the burden in female populations surpassed that in male populations. Furthermore, health inequalities have continued to widen, as evidenced by the significantly expanded gap between high and low SDI regions over the past three decades. Looking ahead, both the absolute economic burden and the share of GDP attributable to TBL cancer are projected to continue rising. This trajectory implies that the absolute strain on Asia’s fragmented health systems and shrinking labor supply will intensify, with low- and middle-income countries and those undergoing rapid population aging facing even greater challenges.

## Data Availability

The original contributions presented in the study are included in the article/Supplementary material, further inquiries can be directed to the corresponding authors.
